# 5-(Naphthalen-1-yl)isophthalic acid–dimethyl sulfoxide–water (2/1/2)

**DOI:** 10.1107/S1600536813012361

**Published:** 2013-05-18

**Authors:** Antje Vetter, Wilhelm Seichter, Edwin Weber

**Affiliations:** a Institut für Organische Chemie, TU Bergakademie Freiberg, Leipziger Strasse 29, D-09596 Freiberg/Sachsen, Germany

## Abstract

The asymmetric unit of the title compound, 2C_18_H_12_O_4_·C_2_H_6_OS·2H_2_O, consists of four crystallographically independent mol­ecules of 5-(naphthalen-1-yl)isophthalic acid, two dimethyl sulfoxide and four water mol­ecules. The dihedral angles formed by the the planes of the aromatic fragments of the organic mol­ecules range from 57.4 (1) to 59.1 (1)°. In the crystal, multiple O—H⋯O hydrogen bonds link the water mol­ecules with the carbonyl and sulfoxide groups, giving rise to double ribbons along the *b-*axis direction.

## Related literature

For preparative methods used for the synthesis of the title compound, see: Broutin & Colobert (2005 ▶); Mazik & König (2006 ▶); Miyaura *et al.* (1981 ▶). For the structure of isophthalic acid, see: Derissen (1974 ▶). For hydrogen-bonding patterns, see: Bernstein *et al.* (1995 ▶); Burrows (2004 ▶). For π–π stacking inter­actions, see: James (2004 ▶). For C—H⋯O inter­actions, see: Desiraju & Steiner (1999 ▶). For organic crystal engineering aspects, see: Tiekink *et al.* (2010 ▶).
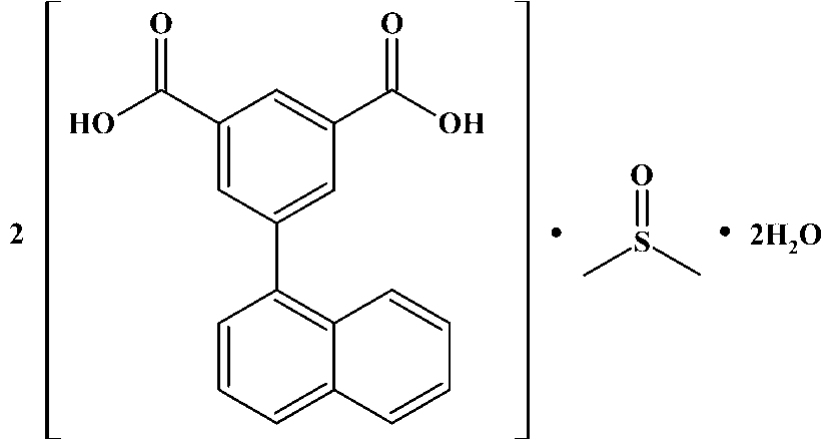


## Experimental

### 

#### Crystal data

2C_18_H_12_O_4_·C_2_H_6_OS·2H_2_O
*M* *_r_* = 698.72
Triclinic, 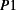

*a* = 6.6842 (4) Å
*b* = 9.6173 (6) Å
*c* = 25.4682 (15) Å
α = 95.780 (3)°
β = 95.669 (3)°
γ = 90.028 (3)°
*V* = 1620.82 (17) Å^3^
*Z* = 2
Mo *K*α radiation
μ = 0.17 mm^−1^
*T* = 93 K
0.18 × 0.17 × 0.09 mm

#### Data collection

Bruker Kappa APEXII CCD diffractometer
39842 measured reflections
15284 independent reflections
12600 reflections with *I* > 2σ(*I*)
*R*_int_ = 0.043

#### Refinement

*R*[*F* ^2^ > 2σ(*F* ^2^)] = 0.045
*wR*(*F* ^2^) = 0.100
*S* = 1.04
15284 reflections
945 parameters
8 restraints
H atoms treated by a mixture of independent and constrained refinement
Δρ_max_ = 0.31 e Å^−3^
Δρ_min_ = −0.29 e Å^−3^
Absolute structure: Flack (1983 ▶), 7387 Friedel pairs
Flack parameter: −0.03 (5)

### 

Data collection: *APEX2* (Bruker, 2007 ▶); cell refinement: *SAINT* (Bruker, 2007 ▶); data reduction: *SAINT*; program(s) used to solve structure: *SHELXS97* (Sheldrick, 2008 ▶); program(s) used to refine structure: *SHELXL97* (Sheldrick, 2008 ▶); molecular graphics: *ORTEP-3 for Windows* (Farrugia, 2012 ▶); software used to prepare material for publication: *SHELXTL* (Sheldrick, 2008 ▶).

## Supplementary Material

Crystal structure: contains datablock(s) I, global. DOI: 10.1107/S1600536813012361/yk2092sup1.cif

Structure factors: contains datablock(s) I. DOI: 10.1107/S1600536813012361/yk2092Isup2.hkl

Supplementary material file. DOI: 10.1107/S1600536813012361/yk2092Isup3.cml

Additional supplementary materials:  crystallographic information; 3D view; checkCIF report

## Figures and Tables

**Table 1:** Hydrogen-bond geometry (Å, °)

*D*—H⋯*A*	*D*—H	H⋯*A*	*D*⋯*A*	*D*—H⋯*A*
O1—H1⋯O1*W* ^i^	0.84	1.72	2.559 (2)	175
O1*B*—H1*B*⋯O3*W*	0.84	1.74	2.573 (2)	168
O2*A*—H2*A*⋯O4*A* ^ii^	0.84	1.82	2.583 (2)	151
O2*C*—H2*C*⋯O4*C* ^ii^	0.84	1.81	2.584 (2)	152
O3*A*—H3*A*⋯O2*W* ^iii^	0.84	1.73	2.565 (3)	174
O3*C*—H3*C*⋯O4*W*	0.84	1.72	2.561 (2)	176
O4—H4⋯O2^iii^	0.84	1.81	2.591 (2)	154
O4*B*—H4*B*⋯O2*B* ^iii^	0.84	1.81	2.578 (2)	150
O1*W*—H1*W*1⋯O1*H* ^iv^	0.85 (3)	1.92 (3)	2.716 (3)	155 (3)
O1*W*—H2*W*1⋯O3^v^	0.85 (3)	2.04 (3)	2.858 (2)	162 (3)
O2*W*—H1*W*2⋯O1*G* ^vi^	0.85 (3)	1.91 (3)	2.742 (3)	168 (3)
O2*W*—H2*W*2⋯O1*A*	0.85 (2)	2.03 (2)	2.879 (2)	174 (3)
O3*W*—H1*W*3⋯O1*G* ^vii^	0.85 (2)	1.85 (2)	2.680 (3)	165 (3)
O3*W*—H2*W*3⋯O3*B* ^ii^	0.85 (2)	1.98 (2)	2.824 (2)	170 (3)
O4*W*—H1*W*4⋯O1*H* ^vii^	0.85 (2)	1.84 (2)	2.656 (3)	162 (3)
O4*W*—H2*W*4⋯O1*C* ^iii^	0.86 (3)	1.96 (3)	2.808 (2)	172 (3)

Symmetry codes: (i) 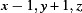; (ii) 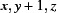; (iii) 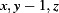; (iv) 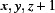; (v) 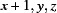; (vi) 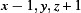; (vii) 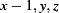.

**Fig. 1.**
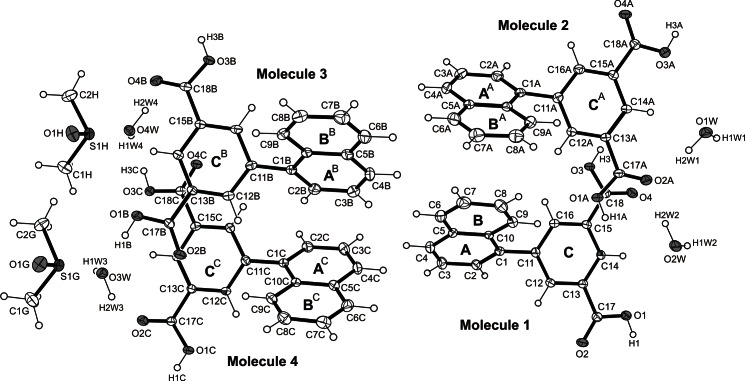
Asymmetric unit of the title compound, showing the atom numbering scheme. Displacement ellipsoids are drawn at the 50% probability level.

**Fig. 2.**
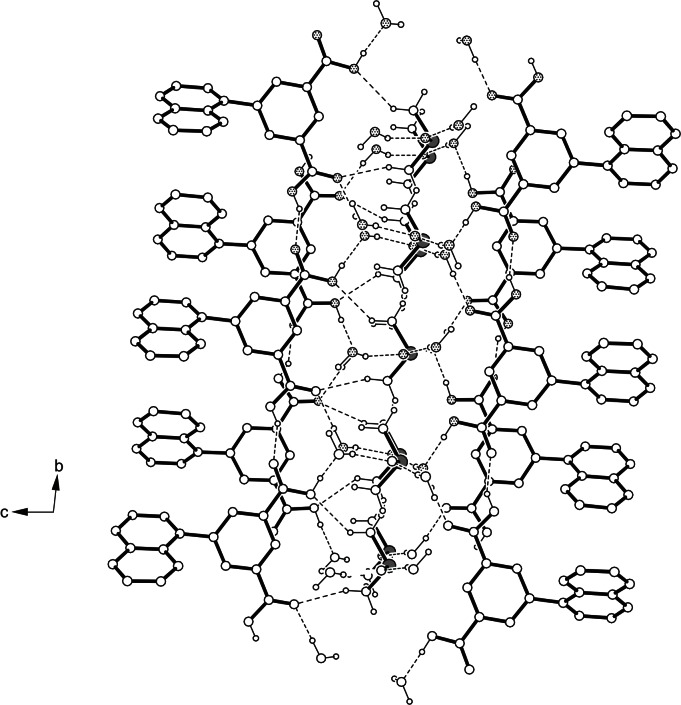
A view along the a-axis of the crystal packing of the title compound. Hydrogen bond interactions are presented as broken lines. Oxygen atoms are specified as dotted circles, sulfur atoms as shaded circles. Non-relevant hydrogen atoms are omitted.

## supplementary crystallographic information

### Comment

In the solid state, isophthalic acid creates an interesting bond stabilized tape structure (Derissen, 1974) with the dimer motif of carboxylic acids (Burrows, 2004) showing particular effectiveness. Substitution of the isophthalic acid with a naphthalene unit that may activate competing π-stacking behaviour in the supramolecular organization (James, 2004) is a challenging question regarding aspects of crystal engineering (Tiekink *et al.*, 2010). This led us to study the crystal structure of a corresponding compound which proved to be a mixed DMSO solvate-hydrate species of 2:1:2 (compound:DMSO:H_2_O) stoichiometry containing four crystallographically independent compound molecules, two DMSO and four water molecules in the asymmetric part of the unit cell (Fig. 1). Regarding the conformation of the naphthyl substituted isophthalic acid molecule, the dihedral angles formed by the planes of the aromatic moieties range from 57.4 (1) to 59.1 (1)°; the isophthalic acid fragments of the molecules are approximately planar. Due to the distinctive donor/acceptor character of the crystal components, the solid phase structure of the title compound is characterized by a complicated pattern of non-covalent intermolecular bonding. The crystal can be regarded as being constructed of molecular double layers extending parallel to the crystallographic *ab* plane (Fig. 2). The aromatic parts of the isophthalic acid molecules form the hydrophobic peripheral areas of the double layer structure, whereas the core region is defined by the polar molecule parts and solvent molecules. In this arrangement, the carboxyl groups and water molecules take part in formation of 10-membered cyclic motifs of O—H···O bonds [*d*(H···O) 1.81–1.82 Å]. Hence, instead of the conventional carboxylic acid dimer of the graph set *R*_2_^2^(8) (Bernstein *et al.*, 1995), an expanded dimer following the graph set *R*_3_^3^(10) is formed. Furthermore, the second hydrogen of each water molecule is engaged in coordination with DMSO molecules, the O atoms of which act as bifurcated acceptors [*d*(H···O) 1.85 (2)–1.92 (3) Å]. A large number of relatively strong non- conventional hydrogen bonds of the C—H···O type (Desiraju & Steiner, 1999) [*d*(H···O) 2.47 (2)–2.55 (3) Å] involving carboxylic and water O atoms complete the network of intermolecular interactions. Within the hydrophobic layer domains, the naphthyl residues of the molecules adopt a herringbone pattern, so that no marked arene based interlayer interactions can be observed. Consequently, only weak van der Waals forces stabilize the crystal packing in direction of the *c*-axis.

### Experimental

Preparation of the title compound was achieved by a Suzuki cross coupling reaction (Miyaura *et al.*, 1981) between 2-(naphthalen-1-yl)-1,3,2- dioxaborolane (Broutin & Colobert, 2005) (3.1 g, 9.5 mmol) in the presence of palladium(II) acetate (217 mg, 0.97 mmol) and potassium phosphate (3.0 g, 14.1 mmol) in 70 ml degassed tetrahydrofuran. The resulting mixture was heated to reflux for 6 h, then cooled to room temperature, quenched with water and filtered through celite. The aqueous phase was extracted with dichloromethane and dried over Na_2_SO_4_. Evaporation of the solvent and crystallization from ethanol yielded 1.20 g (41%) colourless needles with m.p. 421–422 K of the intermediate diester. This diester and powdered sodium hydroxid (5.0 g, 125 mmol) in methanol-water (50 ml, 1:1, *v*/*v*) was refluxed for 4 h. After cooling to room temperature, the mixture was filtered and the filtrate acidified with aqueous hydrochloric acid. The precipitate which has formed was collected by suction filtration, washed with water, dissolved in ethanol-chloroform (1:1) and dried (Na_2_SO_4_). Evaporation of the solvent and crystallization from ethanol yielded 1.1 g (40%) of colourless crystals; m.p. > 593 K. IR (KBr) 3063, 2029, 1848, 1706, 1628, 1603, 805, 779, 763. ^1^H NMR (400 MHz, D_6_–DMSO) 7.54 - 7.66 (m, 4 H, naphthyl-H), 7.75 (d, ^3^J_HH_ = 8 Hz, 1 H, naphthyl-H), 8.05 (m, 2 H, naphthyl-H), 8.23 (s, 2 H, isophthalic acid-H), 8.59 (s, 1 H, isophthalic acid-H), 13.33 (br, 2 H, COOH). ^13^C NMR (100.6 MHz, D_6_–DMSO) 124.7, 125.7, 126.3, 127.0, 127.4, 128.6, 128.7, 129.0, 130.6, 131.8, 133.5, 134.3, 137.6, 140.8, 166.5 (COOH). MS (EI) *m/z*: found - 292.1; calc. for C_18_H_12_O_4_ - 292.25. Palladium(II) acetate was purchased from Aldrich. Melting point was measured on a hot stage microscope (Rapido, Dresden). IR, NMR (^1^H, ^13^C) and mass (EI–MS) spectra were performed using Nicolet 510 FT–IR, Bruker Avance DPX 400 and Finnigan Mat 8200 instruments, respectively. Crystals of the title compound (DMSO solvate-dihydrate) suitable for X-ray structural analysis were grown by slow evaporating a solution of the material described above.

### Refinement

Aromatic H atoms were positioned geometrically and allowed to ride on their parent atoms, with C—H = 0.95 Å and *U*_iso_ = 1.2 *U*_eq_(C).

### Crystal data

**Table:**

2C_18_H_12_O_4_·C_2_H_6_OS·2H_2_O	*Z* = 2
*M**_r_* = 698.72	*F*(000) = 732
Triclinic, *P*1	*D*_x_ = 1.432 Mg m^−^^3^
Hall symbol: P 1	Mo *K*α radiation, λ = 0.71073 Å
*a* = 6.6842 (4) Å	Cell parameters from 8379 reflections
*b* = 9.6173 (6) Å	θ = 2.2–30.6°
*c* = 25.4682 (15) Å	µ = 0.17 mm^−^^1^
α = 95.780 (3)°	*T* = 93 K
β = 95.669 (3)°	Rhombus, colourless
γ = 90.028 (3)°	0.18 × 0.17 × 0.09 mm
*V* = 1620.82 (17) Å^3^	

### Data collection

**Table:**

Bruker Kappa APEXII CCD diffractometer	12600 reflections with *I* > 2σ(*I*)
Radiation source: fine-focus sealed tube	*R*_int_ = 0.043
Graphite monochromator	θ_max_ = 28.1°, θ_min_ = 1.6°
φ and ω scans	*h* = −8→8
39842 measured reflections	*k* = −12→12
15284 independent reflections	*l* = −33→33

### Refinement

**Table:**

Refinement on *F*^2^	Secondary atom site location: difference Fourier map
Least-squares matrix: full	Hydrogen site location: inferred from neighbouring sites
*R*[*F*^2^ > 2σ(*F*^2^)] = 0.045	H atoms treated by a mixture of independent and constrained refinement
*wR*(*F*^2^) = 0.100	*w* = 1/[σ^2^(*F*_o_^2^) + (0.0447*P*)^2^ + 0.2447*P*] where *P* = (*F*_o_^2^ + 2*F*_c_^2^)/3
*S* = 1.04	(Δ/σ)_max_ < 0.001
15284 reflections	Δρ_max_ = 0.31 e Å^−^^3^
945 parameters	Δρ_min_ = −0.29 e Å^−^^3^
8 restraints	Absolute structure: Flack (1983), 7387 Friedel pairs
Primary atom site location: structure-invariant direct methods	Flack parameter: −0.03 (5)

### Special details

**Table:**

Geometry. All s.u.'s (except the s.u. in the dihedral angle between two l.s. planes) are estimated using the full covariance matrix. The cell s.u.'s are taken into account individually in the estimation of s.u.'s in distances, angles and torsion angles; correlations between s.u.'s in cell parameters are only used when they are defined by crystal symmetry. An approximate (isotropic) treatment of cell s.u.'s is used for estimating s.u.'s involving l.s. planes.
Refinement. Refinement of *F*^2^ against ALL reflections. The weighted *R*-factor *wR* and goodness of fit *S* are based on *F*^2^, conventional *R*-factors *R* are based on *F*, with *F* set to zero for negative *F*^2^. The threshold expression of *F*^2^ > σ(*F*^2^) is used only for calculating *R*-factors(gt) *etc*. and is not relevant to the choice of reflections for refinement. *R*-factors based on *F*^2^ are statistically about twice as large as those based on *F*, and *R*- factors based on ALL data will be even larger. Distances for O—H bonds of the water molecules were restraint of 0.85 (0.01) Angstroms.

### Fractional atomic coordinates and isotropic or equivalent isotropic displacement parameters (Å^2^)

**Table:**

	*x*	*y*	*z*	*U*_iso_*/*U*_eq_	
O1	−0.0444 (3)	1.15517 (17)	0.93514 (7)	0.0183 (4)	
H1	−0.0530	1.2373	0.9494	0.027*	
O2	0.0285 (3)	1.26400 (17)	0.86570 (7)	0.0166 (4)	
O3	−0.0263 (3)	0.63317 (18)	0.92376 (7)	0.0181 (4)	
O4	0.0604 (3)	0.52281 (17)	0.84729 (7)	0.0173 (4)	
H4	0.0412	0.4509	0.8624	0.026*	
C1	0.1036 (4)	0.8755 (2)	0.71638 (9)	0.0131 (5)	
C2	−0.0413 (4)	0.9361 (3)	0.68468 (10)	0.0160 (5)	
H2	−0.1476	0.9847	0.7004	0.019*	
C3	−0.0365 (4)	0.9282 (3)	0.62936 (10)	0.0179 (5)	
H3	−0.1383	0.9716	0.6081	0.021*	
C4	0.1153 (4)	0.8578 (3)	0.60616 (10)	0.0171 (5)	
H4A	0.1160	0.8505	0.5687	0.021*	
C5	0.2709 (4)	0.7959 (2)	0.63727 (10)	0.0145 (5)	
C6	0.4338 (4)	0.7260 (3)	0.61436 (10)	0.0185 (5)	
H6	0.4354	0.7172	0.5769	0.022*	
C7	0.5862 (4)	0.6718 (3)	0.64457 (11)	0.0217 (6)	
H7	0.6929	0.6251	0.6283	0.026*	
C8	0.5864 (4)	0.6849 (3)	0.70067 (10)	0.0193 (6)	
H8	0.6946	0.6481	0.7219	0.023*	
C9	0.4319 (4)	0.7502 (2)	0.72416 (10)	0.0158 (5)	
H9	0.4346	0.7587	0.7617	0.019*	
C10	0.2673 (4)	0.8055 (2)	0.69350 (10)	0.0130 (5)	
C11	0.0816 (4)	0.8818 (2)	0.77422 (10)	0.0130 (5)	
C12	0.0566 (4)	1.0103 (2)	0.80377 (9)	0.0133 (5)	
H12	0.0621	1.0943	0.7872	0.016*	
C13	0.0237 (4)	1.0165 (2)	0.85705 (10)	0.0120 (5)	
C14	0.0126 (4)	0.8948 (2)	0.88178 (10)	0.0131 (5)	
H14	−0.0120	0.8991	0.9180	0.016*	
C15	0.0379 (4)	0.7662 (2)	0.85273 (10)	0.0130 (5)	
C16	0.0729 (4)	0.7607 (2)	0.79979 (10)	0.0136 (5)	
H16	0.0914	0.6726	0.7805	0.016*	
C17	0.0031 (4)	1.1579 (3)	0.88623 (10)	0.0140 (5)	
C18	0.0212 (4)	0.6353 (2)	0.87888 (10)	0.0132 (5)	
O1A	0.4749 (3)	0.58077 (17)	0.92172 (7)	0.0176 (4)	
O2A	0.5635 (3)	0.65134 (17)	0.84584 (7)	0.0172 (4)	
H2A	0.5454	0.7311	0.8613	0.026*	
O3A	0.4594 (3)	0.06583 (18)	0.93111 (7)	0.0174 (4)	
H3A	0.4520	−0.0088	0.9454	0.026*	
O4A	0.5295 (3)	−0.08045 (17)	0.86125 (7)	0.0169 (4)	
C1A	0.5968 (4)	0.2372 (2)	0.71184 (10)	0.0142 (5)	
C2A	0.4496 (4)	0.1661 (3)	0.67782 (10)	0.0166 (5)	
H2A1	0.3429	0.1219	0.6920	0.020*	
C3A	0.4543 (4)	0.1576 (3)	0.62245 (10)	0.0196 (6)	
H3A1	0.3517	0.1077	0.5997	0.024*	
C4A	0.6062 (4)	0.2212 (3)	0.60148 (10)	0.0179 (5)	
H4A1	0.6051	0.2186	0.5641	0.022*	
C5A	0.7651 (4)	0.2910 (2)	0.63458 (10)	0.0153 (5)	
C6A	0.9278 (4)	0.3532 (3)	0.61336 (11)	0.0211 (6)	
H6A	0.9287	0.3503	0.5760	0.025*	
C7A	1.0827 (4)	0.4170 (3)	0.64568 (11)	0.0229 (6)	
H7A	1.1897	0.4593	0.6309	0.027*	
C8A	1.0839 (4)	0.4202 (3)	0.70124 (11)	0.0204 (6)	
H8A	1.1940	0.4625	0.7236	0.024*	
C9A	0.9294 (4)	0.3635 (3)	0.72307 (10)	0.0168 (5)	
H9A	0.9330	0.3672	0.7606	0.020*	
C10A	0.7626 (4)	0.2987 (2)	0.69084 (10)	0.0135 (5)	
C11A	0.5760 (4)	0.2556 (2)	0.77016 (10)	0.0128 (5)	
C12A	0.5691 (4)	0.3900 (2)	0.79653 (10)	0.0136 (5)	
H12A	0.5871	0.4688	0.7777	0.016*	
C13A	0.5362 (4)	0.4105 (2)	0.84980 (10)	0.0127 (5)	
C14A	0.5131 (4)	0.2966 (2)	0.87837 (10)	0.0131 (5)	
H14A	0.4905	0.3103	0.9148	0.016*	
C15A	0.5235 (4)	0.1616 (2)	0.85266 (10)	0.0128 (5)	
C16A	0.5526 (4)	0.1418 (2)	0.79892 (10)	0.0140 (5)	
H16A	0.5566	0.0496	0.7817	0.017*	
C17A	0.5220 (4)	0.5552 (2)	0.87681 (10)	0.0135 (5)	
C18A	0.5046 (4)	0.0373 (2)	0.88190 (10)	0.0133 (5)	
O1B	0.6696 (3)	0.72004 (17)	0.17136 (7)	0.0164 (4)	
H1B	0.6567	0.7946	0.1568	0.025*	
O2B	0.7921 (3)	0.86549 (17)	0.24144 (7)	0.0156 (4)	
O3B	0.6950 (3)	0.20477 (17)	0.17970 (7)	0.0165 (4)	
O4B	0.8369 (3)	0.13335 (17)	0.25592 (7)	0.0158 (4)	
H4B	0.7978	0.0542	0.2416	0.024*	
C1B	0.9745 (4)	0.5465 (2)	0.39000 (9)	0.0132 (5)	
C2B	0.8542 (4)	0.6176 (3)	0.42408 (10)	0.0164 (5)	
H2B	0.7367	0.6619	0.4100	0.020*	
C3B	0.9013 (4)	0.6265 (3)	0.47989 (11)	0.0197 (6)	
H3B	0.8162	0.6767	0.5027	0.024*	
C4B	1.0684 (4)	0.5630 (3)	0.50072 (10)	0.0179 (5)	
H4B1	1.0961	0.5658	0.5381	0.022*	
C5B	1.2014 (4)	0.4930 (3)	0.46748 (10)	0.0163 (5)	
C6B	1.3807 (4)	0.4316 (3)	0.48856 (11)	0.0197 (6)	
H6B	1.4107	0.4350	0.5259	0.024*	
C7B	1.5110 (4)	0.3676 (3)	0.45596 (11)	0.0213 (6)	
H7B	1.6296	0.3260	0.4708	0.026*	
C8B	1.4708 (4)	0.3630 (3)	0.40047 (11)	0.0188 (6)	
H8B	1.5632	0.3194	0.3781	0.023*	
C9B	1.2992 (4)	0.4208 (2)	0.37855 (10)	0.0156 (5)	
H9B	1.2746	0.4179	0.3411	0.019*	
C10B	1.1573 (4)	0.4853 (2)	0.41101 (9)	0.0135 (5)	
C11B	0.9094 (4)	0.5295 (2)	0.33200 (9)	0.0122 (5)	
C12B	0.8653 (4)	0.6428 (2)	0.30312 (10)	0.0136 (5)	
H12B	0.8844	0.7351	0.3201	0.016*	
C13B	0.7935 (3)	0.6225 (2)	0.24962 (10)	0.0110 (5)	
C14B	0.7634 (3)	0.4884 (2)	0.22378 (10)	0.0114 (5)	
H14B	0.7123	0.4751	0.1875	0.014*	
C15B	0.8097 (4)	0.3739 (2)	0.25216 (10)	0.0118 (5)	
C16B	0.8828 (4)	0.3948 (2)	0.30538 (10)	0.0130 (5)	
H16B	0.9154	0.3161	0.3242	0.016*	
C17B	0.7521 (4)	0.7479 (2)	0.22060 (9)	0.0116 (5)	
C18B	0.7735 (4)	0.2302 (2)	0.22478 (10)	0.0132 (5)	
O1C	0.1944 (3)	1.14760 (17)	0.17794 (7)	0.0168 (4)	
O2C	0.3329 (3)	1.25902 (17)	0.25475 (7)	0.0160 (4)	
H2C	0.3049	1.3305	0.2391	0.024*	
O3C	0.1643 (3)	0.62594 (17)	0.16752 (6)	0.0153 (4)	
H3C	0.1382	0.5435	0.1544	0.023*	
O4C	0.2889 (3)	0.51794 (17)	0.23747 (7)	0.0157 (4)	
C1C	0.4779 (4)	0.9086 (2)	0.38610 (9)	0.0123 (5)	
C2C	0.3567 (4)	0.8484 (2)	0.41795 (10)	0.0158 (5)	
H2C1	0.2377	0.8004	0.4025	0.019*	
C3C	0.4056 (4)	0.8562 (3)	0.47333 (10)	0.0182 (6)	
H3C1	0.3208	0.8122	0.4947	0.022*	
C4C	0.5741 (4)	0.9268 (3)	0.49644 (10)	0.0170 (5)	
H4C	0.6027	0.9346	0.5339	0.020*	
C5C	0.7072 (4)	0.9887 (3)	0.46510 (10)	0.0147 (5)	
C6C	0.8870 (4)	1.0585 (3)	0.48780 (10)	0.0176 (5)	
H6C	0.9179	1.0672	0.5252	0.021*	
C7C	1.0161 (4)	1.1134 (3)	0.45698 (11)	0.0200 (6)	
H7C	1.1345	1.1612	0.4730	0.024*	
C8C	0.9738 (4)	1.0991 (3)	0.40126 (10)	0.0181 (5)	
H8C	1.0659	1.1353	0.3798	0.022*	
C9C	0.8014 (4)	1.0337 (2)	0.37795 (10)	0.0150 (5)	
H9C	0.7752	1.0251	0.3404	0.018*	
C10C	0.6601 (4)	0.9781 (2)	0.40893 (9)	0.0123 (5)	
C11C	0.4120 (3)	0.9006 (2)	0.32818 (9)	0.0119 (5)	
C12C	0.3846 (4)	1.0224 (2)	0.30269 (9)	0.0117 (5)	
H12C	0.4178	1.1106	0.3219	0.014*	
C13C	0.3094 (4)	1.0156 (2)	0.24968 (10)	0.0115 (5)	
C14C	0.2617 (3)	0.8869 (2)	0.22096 (9)	0.0111 (5)	
H14C	0.2086	0.8822	0.1848	0.013*	
C15C	0.2925 (3)	0.7659 (2)	0.24564 (10)	0.0109 (5)	
C16C	0.3660 (4)	0.7733 (2)	0.29906 (9)	0.0121 (5)	
H16C	0.3847	0.6896	0.3157	0.015*	
C17C	0.2729 (4)	1.1463 (2)	0.22324 (10)	0.0127 (5)	
C18C	0.2487 (4)	0.6250 (2)	0.21663 (10)	0.0124 (5)	
S1	1.00718 (9)	0.89723 (6)	0.08375 (3)	0.01409 (13)	
O1G	1.2225 (3)	0.88624 (19)	0.06914 (7)	0.0208 (4)	
C1G	0.8946 (4)	1.0283 (3)	0.04598 (11)	0.0233 (6)	
H1G1	0.9449	1.1206	0.0615	0.035*	
H1G2	0.7483	1.0243	0.0463	0.035*	
H1G3	0.9288	1.0119	0.0093	0.035*	
C2G	0.8784 (4)	0.7491 (3)	0.04824 (11)	0.0208 (6)	
H2G1	0.9071	0.7420	0.0111	0.031*	
H2G2	0.7334	0.7594	0.0501	0.031*	
H2G3	0.9239	0.6643	0.0640	0.031*	
S2	0.50038 (9)	0.40349 (6)	0.08343 (3)	0.01460 (13)	
O1H	0.7177 (3)	0.40828 (19)	0.07026 (7)	0.0206 (4)	
C1H	0.3754 (4)	0.5325 (3)	0.04717 (11)	0.0221 (6)	
H1H1	0.4134	0.6257	0.0645	0.033*	
H1H2	0.2297	0.5194	0.0462	0.033*	
H1H3	0.4145	0.5234	0.0109	0.033*	
C2H	0.3935 (4)	0.2518 (3)	0.04457 (11)	0.0243 (6)	
H2H1	0.4359	0.2477	0.0087	0.036*	
H2H2	0.2465	0.2563	0.0427	0.036*	
H2H3	0.4393	0.1680	0.0610	0.036*	
O1W	0.9185 (3)	0.40010 (18)	0.98237 (7)	0.0207 (4)	
H1W1	0.829 (4)	0.414 (4)	1.0040 (12)	0.047 (11)*	
H2W1	0.907 (5)	0.466 (3)	0.9622 (11)	0.046 (11)*	
O2W	0.4243 (3)	0.84838 (19)	0.98024 (7)	0.0217 (4)	
H1W2	0.349 (5)	0.853 (4)	1.0051 (11)	0.061 (13)*	
H2W2	0.431 (5)	0.7675 (18)	0.9634 (12)	0.041 (10)*	
O3W	0.5777 (3)	0.94182 (17)	0.12596 (7)	0.0151 (4)	
H1W3	0.458 (2)	0.936 (3)	0.1116 (11)	0.029 (9)*	
H2W3	0.598 (5)	1.0221 (18)	0.1433 (12)	0.039 (10)*	
O4W	0.0735 (3)	0.37916 (18)	0.12416 (7)	0.0155 (4)	
H1W4	−0.048 (2)	0.375 (3)	0.1109 (12)	0.036 (10)*	
H2W4	0.113 (5)	0.314 (3)	0.1433 (11)	0.037 (10)*	

### Atomic displacement parameters (Å^2^)

**Table:**

	*U*^11^	*U*^22^	*U*^33^	*U*^12^	*U*^13^	*U*^23^
O1	0.0302 (11)	0.0112 (8)	0.0143 (9)	0.0033 (8)	0.0068 (8)	0.0009 (7)
O2	0.0228 (10)	0.0100 (8)	0.0183 (10)	0.0005 (7)	0.0067 (8)	0.0029 (7)
O3	0.0258 (10)	0.0152 (9)	0.0147 (9)	0.0012 (7)	0.0065 (8)	0.0042 (7)
O4	0.0285 (11)	0.0090 (8)	0.0152 (9)	−0.0001 (7)	0.0053 (8)	0.0021 (7)
C1	0.0156 (13)	0.0102 (11)	0.0133 (12)	−0.0025 (9)	0.0007 (10)	0.0005 (9)
C2	0.0163 (13)	0.0149 (12)	0.0172 (13)	0.0022 (10)	0.0021 (10)	0.0032 (10)
C3	0.0194 (14)	0.0194 (13)	0.0147 (13)	0.0004 (10)	−0.0020 (11)	0.0049 (10)
C4	0.0209 (14)	0.0188 (13)	0.0118 (12)	−0.0010 (10)	0.0019 (10)	0.0026 (10)
C5	0.0206 (13)	0.0107 (11)	0.0124 (12)	−0.0034 (9)	0.0036 (10)	0.0003 (9)
C6	0.0240 (14)	0.0179 (13)	0.0144 (13)	−0.0015 (11)	0.0066 (11)	0.0012 (10)
C7	0.0252 (15)	0.0174 (13)	0.0240 (14)	0.0026 (11)	0.0096 (12)	0.0014 (11)
C8	0.0167 (13)	0.0194 (13)	0.0229 (14)	0.0034 (10)	0.0010 (11)	0.0076 (11)
C9	0.0183 (13)	0.0152 (12)	0.0141 (13)	−0.0008 (10)	0.0009 (10)	0.0026 (10)
C10	0.0188 (13)	0.0080 (11)	0.0128 (12)	−0.0031 (9)	0.0032 (10)	0.0025 (9)
C11	0.0129 (12)	0.0140 (12)	0.0125 (12)	0.0000 (9)	0.0027 (9)	0.0029 (9)
C12	0.0146 (12)	0.0109 (11)	0.0142 (12)	−0.0007 (9)	−0.0015 (10)	0.0035 (9)
C13	0.0103 (12)	0.0109 (11)	0.0144 (12)	0.0002 (9)	−0.0004 (9)	0.0010 (9)
C14	0.0164 (13)	0.0131 (12)	0.0100 (12)	0.0003 (9)	0.0019 (10)	0.0015 (9)
C15	0.0147 (12)	0.0104 (11)	0.0142 (12)	−0.0001 (9)	0.0015 (10)	0.0026 (9)
C16	0.0165 (13)	0.0090 (11)	0.0149 (13)	0.0004 (9)	0.0007 (10)	0.0004 (9)
C17	0.0127 (12)	0.0151 (12)	0.0146 (13)	0.0024 (9)	0.0034 (10)	0.0015 (10)
C18	0.0115 (12)	0.0114 (12)	0.0157 (13)	−0.0011 (9)	−0.0013 (10)	−0.0005 (9)
O1A	0.0253 (10)	0.0128 (9)	0.0150 (9)	0.0003 (7)	0.0051 (8)	0.0001 (7)
O2A	0.0263 (10)	0.0090 (8)	0.0166 (9)	0.0005 (7)	0.0033 (8)	0.0009 (7)
O3A	0.0263 (10)	0.0132 (9)	0.0135 (9)	−0.0029 (7)	0.0054 (8)	0.0024 (7)
O4A	0.0231 (10)	0.0094 (8)	0.0188 (10)	0.0007 (7)	0.0050 (8)	0.0012 (7)
C1A	0.0187 (13)	0.0103 (11)	0.0139 (12)	0.0025 (9)	0.0025 (10)	0.0015 (9)
C2A	0.0192 (13)	0.0125 (12)	0.0187 (13)	−0.0003 (10)	0.0044 (11)	0.0023 (10)
C3A	0.0232 (15)	0.0151 (13)	0.0187 (14)	0.0004 (11)	−0.0009 (11)	−0.0035 (10)
C4A	0.0270 (15)	0.0154 (12)	0.0110 (12)	0.0035 (10)	0.0028 (11)	−0.0008 (10)
C5A	0.0231 (13)	0.0090 (11)	0.0142 (12)	0.0030 (10)	0.0045 (10)	0.0005 (9)
C6A	0.0259 (15)	0.0195 (13)	0.0198 (14)	0.0033 (11)	0.0091 (12)	0.0041 (11)
C7A	0.0229 (15)	0.0195 (13)	0.0281 (15)	−0.0008 (11)	0.0101 (12)	0.0037 (11)
C8A	0.0176 (14)	0.0176 (13)	0.0261 (15)	0.0015 (10)	0.0040 (11)	0.0007 (11)
C9A	0.0203 (14)	0.0155 (12)	0.0145 (13)	0.0022 (10)	0.0036 (11)	−0.0002 (10)
C10A	0.0183 (13)	0.0086 (11)	0.0142 (12)	0.0041 (9)	0.0033 (10)	0.0020 (9)
C11A	0.0121 (12)	0.0129 (11)	0.0135 (12)	−0.0002 (9)	0.0029 (10)	0.0006 (9)
C12A	0.0163 (13)	0.0097 (11)	0.0143 (12)	−0.0002 (9)	−0.0016 (10)	0.0021 (9)
C13A	0.0123 (12)	0.0097 (11)	0.0160 (13)	0.0011 (9)	0.0008 (10)	0.0013 (9)
C14A	0.0153 (12)	0.0096 (11)	0.0145 (12)	0.0012 (9)	0.0023 (10)	0.0005 (9)
C15A	0.0104 (12)	0.0122 (11)	0.0161 (13)	0.0004 (9)	0.0001 (10)	0.0037 (9)
C16A	0.0128 (12)	0.0100 (11)	0.0189 (13)	−0.0004 (9)	0.0002 (10)	0.0012 (10)
C17A	0.0125 (12)	0.0107 (11)	0.0169 (13)	−0.0008 (9)	−0.0007 (10)	0.0014 (10)
C18A	0.0115 (12)	0.0135 (12)	0.0151 (13)	−0.0006 (9)	0.0005 (10)	0.0034 (10)
O1B	0.0230 (10)	0.0105 (8)	0.0154 (9)	0.0027 (7)	−0.0015 (8)	0.0031 (7)
O2B	0.0195 (9)	0.0092 (8)	0.0177 (9)	0.0001 (7)	−0.0015 (7)	0.0030 (7)
O3B	0.0218 (10)	0.0120 (8)	0.0144 (9)	−0.0008 (7)	−0.0019 (7)	−0.0006 (7)
O4B	0.0255 (10)	0.0075 (8)	0.0137 (9)	−0.0017 (7)	−0.0020 (7)	0.0026 (7)
C1B	0.0178 (13)	0.0073 (11)	0.0143 (12)	−0.0026 (9)	−0.0001 (10)	0.0017 (9)
C2B	0.0182 (13)	0.0125 (12)	0.0185 (13)	0.0022 (10)	0.0003 (10)	0.0021 (10)
C3B	0.0243 (15)	0.0144 (13)	0.0208 (14)	0.0016 (11)	0.0066 (11)	0.0003 (10)
C4B	0.0237 (14)	0.0193 (13)	0.0102 (12)	−0.0040 (11)	−0.0006 (11)	0.0011 (10)
C5B	0.0217 (14)	0.0107 (12)	0.0160 (13)	−0.0042 (10)	−0.0012 (11)	0.0016 (10)
C6B	0.0244 (15)	0.0182 (13)	0.0152 (13)	−0.0029 (11)	−0.0052 (11)	0.0024 (10)
C7B	0.0186 (14)	0.0182 (13)	0.0263 (15)	0.0007 (11)	−0.0045 (11)	0.0046 (11)
C8B	0.0168 (13)	0.0148 (12)	0.0239 (14)	0.0012 (10)	0.0016 (11)	−0.0023 (10)
C9B	0.0176 (13)	0.0122 (12)	0.0161 (13)	−0.0031 (9)	−0.0010 (10)	−0.0006 (10)
C10B	0.0174 (13)	0.0101 (11)	0.0126 (12)	−0.0031 (9)	−0.0001 (10)	0.0009 (9)
C11B	0.0112 (12)	0.0104 (11)	0.0148 (12)	−0.0005 (9)	0.0016 (10)	−0.0002 (9)
C12B	0.0138 (12)	0.0090 (11)	0.0176 (13)	−0.0006 (9)	0.0018 (10)	−0.0003 (9)
C13B	0.0073 (11)	0.0121 (11)	0.0142 (12)	0.0036 (9)	0.0024 (9)	0.0033 (9)
C14B	0.0101 (12)	0.0117 (11)	0.0127 (12)	−0.0011 (9)	0.0011 (9)	0.0030 (9)
C15B	0.0115 (12)	0.0092 (11)	0.0153 (12)	0.0000 (9)	0.0035 (10)	0.0024 (9)
C16B	0.0124 (12)	0.0085 (11)	0.0188 (13)	−0.0004 (9)	0.0030 (10)	0.0029 (9)
C17B	0.0107 (12)	0.0123 (11)	0.0119 (12)	0.0009 (9)	0.0003 (9)	0.0028 (9)
C18B	0.0127 (12)	0.0106 (11)	0.0171 (13)	0.0008 (9)	0.0036 (10)	0.0031 (10)
O1C	0.0223 (10)	0.0131 (9)	0.0146 (9)	0.0004 (7)	−0.0024 (7)	0.0037 (7)
O2C	0.0252 (10)	0.0077 (8)	0.0147 (9)	−0.0009 (7)	−0.0014 (8)	0.0029 (7)
O3C	0.0233 (10)	0.0094 (8)	0.0116 (9)	−0.0027 (7)	−0.0027 (7)	−0.0019 (7)
O4C	0.0209 (10)	0.0095 (8)	0.0163 (9)	−0.0012 (7)	−0.0021 (7)	0.0024 (7)
C1C	0.0167 (13)	0.0075 (10)	0.0126 (12)	0.0016 (9)	0.0008 (10)	0.0010 (9)
C2C	0.0171 (13)	0.0136 (12)	0.0165 (13)	−0.0040 (10)	0.0001 (10)	0.0015 (10)
C3C	0.0213 (14)	0.0180 (13)	0.0171 (13)	−0.0014 (11)	0.0064 (11)	0.0059 (10)
C4C	0.0220 (14)	0.0185 (12)	0.0106 (12)	0.0040 (10)	0.0003 (10)	0.0031 (10)
C5C	0.0167 (13)	0.0126 (12)	0.0149 (13)	0.0021 (10)	0.0003 (10)	0.0036 (10)
C6C	0.0196 (14)	0.0169 (13)	0.0145 (13)	0.0033 (10)	−0.0039 (11)	−0.0010 (10)
C7C	0.0169 (13)	0.0174 (13)	0.0244 (14)	−0.0015 (10)	−0.0047 (11)	0.0029 (11)
C8C	0.0150 (13)	0.0180 (13)	0.0216 (14)	−0.0006 (10)	−0.0008 (11)	0.0058 (11)
C9C	0.0172 (13)	0.0135 (12)	0.0151 (13)	0.0026 (10)	0.0017 (10)	0.0048 (10)
C10C	0.0162 (12)	0.0077 (10)	0.0132 (12)	0.0019 (9)	0.0016 (10)	0.0016 (9)
C11C	0.0110 (12)	0.0130 (11)	0.0121 (12)	0.0011 (9)	0.0019 (9)	0.0030 (9)
C12C	0.0121 (12)	0.0088 (11)	0.0142 (12)	−0.0007 (9)	0.0029 (10)	0.0002 (9)
C13C	0.0110 (12)	0.0106 (11)	0.0134 (12)	0.0001 (9)	0.0041 (9)	0.0009 (9)
C14C	0.0127 (12)	0.0120 (11)	0.0084 (12)	0.0010 (9)	0.0003 (9)	0.0009 (9)
C15C	0.0079 (11)	0.0090 (11)	0.0154 (12)	0.0004 (9)	0.0018 (9)	−0.0010 (9)
C16C	0.0128 (12)	0.0127 (12)	0.0114 (12)	0.0002 (9)	0.0014 (10)	0.0037 (9)
C17C	0.0139 (12)	0.0108 (11)	0.0142 (13)	0.0006 (9)	0.0043 (10)	0.0023 (9)
C18C	0.0128 (12)	0.0105 (11)	0.0137 (12)	−0.0005 (9)	0.0009 (10)	0.0007 (9)
S1	0.0149 (3)	0.0153 (3)	0.0118 (3)	−0.0002 (2)	0.0010 (2)	0.0005 (2)
O1G	0.0136 (9)	0.0261 (10)	0.0220 (10)	−0.0011 (8)	0.0023 (8)	−0.0005 (8)
C1G	0.0254 (15)	0.0225 (14)	0.0245 (15)	0.0029 (11)	0.0071 (12)	0.0105 (11)
C2G	0.0216 (14)	0.0174 (13)	0.0223 (14)	−0.0027 (10)	0.0016 (11)	−0.0037 (11)
S2	0.0140 (3)	0.0170 (3)	0.0130 (3)	0.0012 (2)	0.0017 (2)	0.0019 (2)
O1H	0.0136 (9)	0.0284 (10)	0.0209 (10)	0.0007 (8)	0.0024 (7)	0.0078 (8)
C1H	0.0223 (15)	0.0197 (13)	0.0252 (15)	0.0017 (11)	0.0018 (12)	0.0074 (11)
C2H	0.0253 (15)	0.0157 (13)	0.0312 (16)	−0.0022 (11)	0.0061 (12)	−0.0041 (11)
O1W	0.0348 (12)	0.0127 (9)	0.0159 (10)	0.0017 (8)	0.0092 (9)	0.0007 (8)
O2W	0.0360 (12)	0.0147 (9)	0.0164 (10)	0.0001 (8)	0.0109 (9)	0.0039 (8)
O3W	0.0179 (10)	0.0123 (9)	0.0146 (9)	0.0027 (7)	0.0001 (8)	0.0006 (7)
O4W	0.0173 (10)	0.0152 (9)	0.0140 (9)	−0.0008 (7)	−0.0010 (8)	0.0040 (7)

### Geometric parameters (Å, º)

**Table:**

O1—C17	1.318 (3)	C4B—C5B	1.412 (4)
O1—H1	0.8400	C4B—H4B1	0.9500
O2—C17	1.212 (3)	C5B—C6B	1.418 (4)
O3—C18	1.218 (3)	C5B—C10B	1.434 (3)
O4—C18	1.324 (3)	C6B—C7B	1.367 (4)
O4—H4	0.8400	C6B—H6B	0.9500
C1—C2	1.368 (3)	C7B—C8B	1.408 (4)
C1—C10	1.427 (3)	C7B—H7B	0.9500
C1—C11	1.490 (3)	C8B—C9B	1.369 (4)
C2—C3	1.407 (3)	C8B—H8B	0.9500
C2—H2	0.9500	C9B—C10B	1.421 (3)
C3—C4	1.370 (4)	C9B—H9B	0.9500
C3—H3	0.9500	C11B—C12B	1.391 (3)
C4—C5	1.413 (4)	C11B—C16B	1.403 (3)
C4—H4A	0.9500	C12B—C13B	1.394 (3)
C5—C6	1.423 (3)	C12B—H12B	0.9500
C5—C10	1.428 (3)	C13B—C14B	1.393 (3)
C6—C7	1.352 (4)	C13B—C17B	1.488 (3)
C6—H6	0.9500	C14B—C15B	1.396 (3)
C7—C8	1.422 (4)	C14B—H14B	0.9500
C7—H7	0.9500	C15B—C16B	1.389 (3)
C8—C9	1.365 (4)	C15B—C18B	1.492 (3)
C8—H8	0.9500	C16B—H16B	0.9500
C9—C10	1.421 (4)	O1C—C17C	1.220 (3)
C9—H9	0.9500	O2C—C17C	1.319 (3)
C11—C16	1.395 (3)	O2C—H2C	0.8400
C11—C12	1.399 (3)	O3C—C18C	1.321 (3)
C12—C13	1.391 (3)	O3C—H3C	0.8400
C12—H12	0.9500	O4C—C18C	1.223 (3)
C13—C14	1.389 (3)	C1C—C2C	1.368 (3)
C13—C17	1.496 (3)	C1C—C10C	1.432 (3)
C14—C15	1.396 (3)	C1C—C11C	1.492 (3)
C14—H14	0.9500	C2C—C3C	1.410 (4)
C15—C16	1.387 (3)	C2C—H2C1	0.9500
C15—C18	1.491 (3)	C3C—C4C	1.365 (4)
C16—H16	0.9500	C3C—H3C1	0.9500
O1A—C17A	1.218 (3)	C4C—C5C	1.421 (4)
O2A—C17A	1.321 (3)	C4C—H4C	0.9500
O2A—H2A	0.8400	C5C—C6C	1.419 (4)
O3A—C18A	1.320 (3)	C5C—C10C	1.427 (3)
O3A—H3A	0.8400	C6C—C7C	1.362 (4)
O4A—C18A	1.218 (3)	C6C—H6C	0.9500
C1A—C2A	1.378 (4)	C7C—C8C	1.412 (4)
C1A—C10A	1.430 (4)	C7C—H7C	0.9500
C1A—C11A	1.498 (3)	C8C—C9C	1.365 (4)
C2A—C3A	1.408 (4)	C8C—H8C	0.9500
C2A—H2A1	0.9500	C9C—C10C	1.426 (3)
C3A—C4A	1.365 (4)	C9C—H9C	0.9500
C3A—H3A1	0.9500	C11C—C16C	1.384 (3)
C4A—C5A	1.413 (4)	C11C—C12C	1.399 (3)
C4A—H4A1	0.9500	C12C—C13C	1.388 (3)
C5A—C6A	1.419 (4)	C12C—H12C	0.9500
C5A—C10A	1.429 (3)	C13C—C14C	1.394 (3)
C6A—C7A	1.363 (4)	C13C—C17C	1.493 (3)
C6A—H6A	0.9500	C14C—C15C	1.383 (3)
C7A—C8A	1.412 (4)	C14C—H14C	0.9500
C7A—H7A	0.9500	C15C—C16C	1.395 (3)
C8A—C9A	1.359 (4)	C15C—C18C	1.491 (3)
C8A—H8A	0.9500	C16C—H16C	0.9500
C9A—C10A	1.422 (4)	S1—O1G	1.5229 (18)
C9A—H9A	0.9500	S1—C2G	1.782 (3)
C11A—C16A	1.393 (3)	S1—C1G	1.784 (3)
C11A—C12A	1.399 (3)	C1G—H1G1	0.9800
C12A—C13A	1.390 (3)	C1G—H1G2	0.9800
C12A—H12A	0.9500	C1G—H1G3	0.9800
C13A—C14A	1.391 (3)	C2G—H2G1	0.9800
C13A—C17A	1.496 (3)	C2G—H2G2	0.9800
C14A—C15A	1.399 (3)	C2G—H2G3	0.9800
C14A—H14A	0.9500	S2—O1H	1.5242 (18)
C15A—C16A	1.396 (3)	S2—C1H	1.780 (3)
C15A—C18A	1.483 (3)	S2—C2H	1.785 (3)
C16A—H16A	0.9500	C1H—H1H1	0.9800
O1B—C17B	1.321 (3)	C1H—H1H2	0.9800
O1B—H1B	0.8400	C1H—H1H3	0.9800
O2B—C17B	1.218 (3)	C2H—H2H1	0.9800
O3B—C18B	1.216 (3)	C2H—H2H2	0.9800
O4B—C18B	1.326 (3)	C2H—H2H3	0.9800
O4B—H4B	0.8400	O1W—H1W1	0.856 (10)
C1B—C2B	1.374 (3)	O1W—H2W1	0.856 (10)
C1B—C10B	1.435 (3)	O2W—H1W2	0.846 (10)
C1B—C11B	1.491 (3)	O2W—H2W2	0.853 (10)
C2B—C3B	1.419 (4)	O3W—H1W3	0.847 (10)
C2B—H2B	0.9500	O3W—H2W3	0.853 (10)
C3B—C4B	1.361 (4)	O4W—H1W4	0.849 (10)
C3B—H3B	0.9500	O4W—H2W4	0.861 (10)
			
C17—O1—H1	109.5	C7B—C6B—C5B	121.0 (3)
C18—O4—H4	109.5	C7B—C6B—H6B	119.5
C2—C1—C10	119.8 (2)	C5B—C6B—H6B	119.5
C2—C1—C11	118.2 (2)	C6B—C7B—C8B	120.4 (2)
C10—C1—C11	121.9 (2)	C6B—C7B—H7B	119.8
C1—C2—C3	121.5 (2)	C8B—C7B—H7B	119.8
C1—C2—H2	119.2	C9B—C8B—C7B	120.4 (2)
C3—C2—H2	119.2	C9B—C8B—H8B	119.8
C4—C3—C2	119.8 (2)	C7B—C8B—H8B	119.8
C4—C3—H3	120.1	C8B—C9B—C10B	121.0 (2)
C2—C3—H3	120.1	C8B—C9B—H9B	119.5
C3—C4—C5	120.8 (2)	C10B—C9B—H9B	119.5
C3—C4—H4A	119.6	C9B—C10B—C5B	118.4 (2)
C5—C4—H4A	119.6	C9B—C10B—C1B	123.2 (2)
C4—C5—C6	122.0 (2)	C5B—C10B—C1B	118.4 (2)
C4—C5—C10	119.3 (2)	C12B—C11B—C16B	118.0 (2)
C6—C5—C10	118.7 (2)	C12B—C11B—C1B	122.5 (2)
C7—C6—C5	121.6 (2)	C16B—C11B—C1B	119.5 (2)
C7—C6—H6	119.2	C11B—C12B—C13B	120.8 (2)
C5—C6—H6	119.2	C11B—C12B—H12B	119.6
C6—C7—C8	119.8 (2)	C13B—C12B—H12B	119.6
C6—C7—H7	120.1	C14B—C13B—C12B	120.9 (2)
C8—C7—H7	120.1	C14B—C13B—C17B	120.8 (2)
C9—C8—C7	120.3 (2)	C12B—C13B—C17B	118.3 (2)
C9—C8—H8	119.8	C13B—C14B—C15B	118.9 (2)
C7—C8—H8	119.8	C13B—C14B—H14B	120.6
C8—C9—C10	121.2 (2)	C15B—C14B—H14B	120.6
C8—C9—H9	119.4	C16B—C15B—C14B	120.0 (2)
C10—C9—H9	119.4	C16B—C15B—C18B	121.2 (2)
C9—C10—C1	123.1 (2)	C14B—C15B—C18B	118.9 (2)
C9—C10—C5	118.2 (2)	C15B—C16B—C11B	121.5 (2)
C1—C10—C5	118.6 (2)	C15B—C16B—H16B	119.2
C16—C11—C12	118.0 (2)	C11B—C16B—H16B	119.2
C16—C11—C1	121.4 (2)	O2B—C17B—O1B	123.9 (2)
C12—C11—C1	120.5 (2)	O2B—C17B—C13B	121.6 (2)
C13—C12—C11	120.8 (2)	O1B—C17B—C13B	114.5 (2)
C13—C12—H12	119.6	O3B—C18B—O4B	124.1 (2)
C11—C12—H12	119.6	O3B—C18B—C15B	124.4 (2)
C14—C13—C12	120.5 (2)	O4B—C18B—C15B	111.5 (2)
C14—C13—C17	121.9 (2)	C17C—O2C—H2C	109.5
C12—C13—C17	117.6 (2)	C18C—O3C—H3C	109.5
C13—C14—C15	119.2 (2)	C2C—C1C—C10C	119.8 (2)
C13—C14—H14	120.4	C2C—C1C—C11C	118.0 (2)
C15—C14—H14	120.4	C10C—C1C—C11C	122.2 (2)
C16—C15—C14	120.1 (2)	C1C—C2C—C3C	121.2 (2)
C16—C15—C18	120.7 (2)	C1C—C2C—H2C1	119.4
C14—C15—C18	119.2 (2)	C3C—C2C—H2C1	119.4
C15—C16—C11	121.4 (2)	C4C—C3C—C2C	120.3 (2)
C15—C16—H16	119.3	C4C—C3C—H3C1	119.8
C11—C16—H16	119.3	C2C—C3C—H3C1	119.8
O2—C17—O1	124.2 (2)	C3C—C4C—C5C	120.7 (2)
O2—C17—C13	121.7 (2)	C3C—C4C—H4C	119.6
O1—C17—C13	114.1 (2)	C5C—C4C—H4C	119.6
O3—C18—O4	124.5 (2)	C6C—C5C—C4C	122.0 (2)
O3—C18—C15	123.5 (2)	C6C—C5C—C10C	119.0 (2)
O4—C18—C15	112.0 (2)	C4C—C5C—C10C	118.9 (2)
C17A—O2A—H2A	109.5	C7C—C6C—C5C	121.3 (2)
C18A—O3A—H3A	109.5	C7C—C6C—H6C	119.4
C2A—C1A—C10A	119.6 (2)	C5C—C6C—H6C	119.4
C2A—C1A—C11A	119.6 (2)	C6C—C7C—C8C	120.0 (2)
C10A—C1A—C11A	120.7 (2)	C6C—C7C—H7C	120.0
C1A—C2A—C3A	121.3 (2)	C8C—C7C—H7C	120.0
C1A—C2A—H2A1	119.3	C9C—C8C—C7C	120.5 (2)
C3A—C2A—H2A1	119.3	C9C—C8C—H8C	119.8
C4A—C3A—C2A	120.0 (2)	C7C—C8C—H8C	119.8
C4A—C3A—H3A1	120.0	C8C—C9C—C10C	121.2 (2)
C2A—C3A—H3A1	120.0	C8C—C9C—H9C	119.4
C3A—C4A—C5A	121.0 (2)	C10C—C9C—H9C	119.4
C3A—C4A—H4A1	119.5	C9C—C10C—C5C	118.0 (2)
C5A—C4A—H4A1	119.5	C9C—C10C—C1C	123.0 (2)
C4A—C5A—C6A	121.6 (2)	C5C—C10C—C1C	119.0 (2)
C4A—C5A—C10A	119.3 (2)	C16C—C11C—C12C	118.4 (2)
C6A—C5A—C10A	119.1 (2)	C16C—C11C—C1C	120.8 (2)
C7A—C6A—C5A	121.1 (3)	C12C—C11C—C1C	120.6 (2)
C7A—C6A—H6A	119.4	C13C—C12C—C11C	120.7 (2)
C5A—C6A—H6A	119.4	C13C—C12C—H12C	119.7
C6A—C7A—C8A	119.8 (3)	C11C—C12C—H12C	119.7
C6A—C7A—H7A	120.1	C12C—C13C—C14C	120.3 (2)
C8A—C7A—H7A	120.1	C12C—C13C—C17C	120.5 (2)
C9A—C8A—C7A	120.8 (3)	C14C—C13C—C17C	119.2 (2)
C9A—C8A—H8A	119.6	C15C—C14C—C13C	119.3 (2)
C7A—C8A—H8A	119.6	C15C—C14C—H14C	120.4
C8A—C9A—C10A	121.2 (2)	C13C—C14C—H14C	120.4
C8A—C9A—H9A	119.4	C14C—C15C—C16C	120.2 (2)
C10A—C9A—H9A	119.4	C14C—C15C—C18C	121.7 (2)
C9A—C10A—C1A	123.4 (2)	C16C—C15C—C18C	118.1 (2)
C9A—C10A—C5A	117.9 (2)	C11C—C16C—C15C	121.1 (2)
C1A—C10A—C5A	118.6 (2)	C11C—C16C—H16C	119.5
C16A—C11A—C12A	118.3 (2)	C15C—C16C—H16C	119.5
C16A—C11A—C1A	121.8 (2)	O1C—C17C—O2C	124.4 (2)
C12A—C11A—C1A	119.9 (2)	O1C—C17C—C13C	123.5 (2)
C13A—C12A—C11A	121.2 (2)	O2C—C17C—C13C	112.1 (2)
C13A—C12A—H12A	119.4	O4C—C18C—O3C	123.5 (2)
C11A—C12A—H12A	119.4	O4C—C18C—C15C	121.6 (2)
C12A—C13A—C14A	120.4 (2)	O3C—C18C—C15C	114.9 (2)
C12A—C13A—C17A	120.4 (2)	O1G—S1—C2G	105.32 (11)
C14A—C13A—C17A	119.3 (2)	O1G—S1—C1G	105.23 (12)
C13A—C14A—C15A	119.0 (2)	C2G—S1—C1G	98.15 (13)
C13A—C14A—H14A	120.5	S1—C1G—H1G1	109.5
C15A—C14A—H14A	120.5	S1—C1G—H1G2	109.5
C16A—C15A—C14A	120.4 (2)	H1G1—C1G—H1G2	109.5
C16A—C15A—C18A	118.8 (2)	S1—C1G—H1G3	109.5
C14A—C15A—C18A	120.8 (2)	H1G1—C1G—H1G3	109.5
C11A—C16A—C15A	120.8 (2)	H1G2—C1G—H1G3	109.5
C11A—C16A—H16A	119.6	S1—C2G—H2G1	109.5
C15A—C16A—H16A	119.6	S1—C2G—H2G2	109.5
O1A—C17A—O2A	124.2 (2)	H2G1—C2G—H2G2	109.5
O1A—C17A—C13A	123.8 (2)	S1—C2G—H2G3	109.5
O2A—C17A—C13A	111.9 (2)	H2G1—C2G—H2G3	109.5
O4A—C18A—O3A	124.0 (2)	H2G2—C2G—H2G3	109.5
O4A—C18A—C15A	121.5 (2)	O1H—S2—C1H	104.86 (12)
O3A—C18A—C15A	114.5 (2)	O1H—S2—C2H	104.87 (12)
C17B—O1B—H1B	109.5	C1H—S2—C2H	98.30 (13)
C18B—O4B—H4B	109.5	S2—C1H—H1H1	109.5
C2B—C1B—C10B	119.4 (2)	S2—C1H—H1H2	109.5
C2B—C1B—C11B	119.5 (2)	H1H1—C1H—H1H2	109.5
C10B—C1B—C11B	121.0 (2)	S2—C1H—H1H3	109.5
C1B—C2B—C3B	121.5 (2)	H1H1—C1H—H1H3	109.5
C1B—C2B—H2B	119.2	H1H2—C1H—H1H3	109.5
C3B—C2B—H2B	119.2	S2—C2H—H2H1	109.5
C4B—C3B—C2B	119.9 (2)	S2—C2H—H2H2	109.5
C4B—C3B—H3B	120.0	H2H1—C2H—H2H2	109.5
C2B—C3B—H3B	120.0	S2—C2H—H2H3	109.5
C3B—C4B—C5B	120.9 (2)	H2H1—C2H—H2H3	109.5
C3B—C4B—H4B1	119.6	H2H2—C2H—H2H3	109.5
C5B—C4B—H4B1	119.6	H1W1—O1W—H2W1	106 (3)
C4B—C5B—C6B	121.6 (2)	H1W2—O2W—H2W2	115 (4)
C4B—C5B—C10B	119.7 (2)	H1W3—O3W—H2W3	110 (3)
C6B—C5B—C10B	118.7 (2)	H1W4—O4W—H2W4	117 (3)
			
C10—C1—C2—C3	1.8 (4)	C10B—C1B—C2B—C3B	−3.1 (4)
C11—C1—C2—C3	−176.7 (2)	C11B—C1B—C2B—C3B	174.7 (2)
C1—C2—C3—C4	0.4 (4)	C1B—C2B—C3B—C4B	−0.3 (4)
C2—C3—C4—C5	−1.7 (4)	C2B—C3B—C4B—C5B	2.7 (4)
C3—C4—C5—C6	−178.0 (2)	C3B—C4B—C5B—C6B	177.2 (2)
C3—C4—C5—C10	0.9 (4)	C3B—C4B—C5B—C10B	−1.7 (4)
C4—C5—C6—C7	177.4 (3)	C4B—C5B—C6B—C7B	−178.1 (3)
C10—C5—C6—C7	−1.5 (4)	C10B—C5B—C6B—C7B	0.8 (4)
C5—C6—C7—C8	−0.4 (4)	C5B—C6B—C7B—C8B	0.8 (4)
C6—C7—C8—C9	1.0 (4)	C6B—C7B—C8B—C9B	−0.9 (4)
C7—C8—C9—C10	0.4 (4)	C7B—C8B—C9B—C10B	−0.7 (4)
C8—C9—C10—C1	−179.5 (2)	C8B—C9B—C10B—C5B	2.3 (3)
C8—C9—C10—C5	−2.3 (4)	C8B—C9B—C10B—C1B	−179.5 (2)
C2—C1—C10—C9	174.6 (2)	C4B—C5B—C10B—C9B	176.6 (2)
C11—C1—C10—C9	−7.0 (4)	C6B—C5B—C10B—C9B	−2.3 (3)
C2—C1—C10—C5	−2.5 (3)	C4B—C5B—C10B—C1B	−1.7 (3)
C11—C1—C10—C5	175.9 (2)	C6B—C5B—C10B—C1B	179.4 (2)
C4—C5—C10—C9	−176.1 (2)	C2B—C1B—C10B—C9B	−174.2 (2)
C6—C5—C10—C9	2.8 (3)	C11B—C1B—C10B—C9B	8.1 (4)
C4—C5—C10—C1	1.2 (3)	C2B—C1B—C10B—C5B	4.0 (3)
C6—C5—C10—C1	−179.9 (2)	C11B—C1B—C10B—C5B	−173.7 (2)
C2—C1—C11—C16	121.8 (3)	C2B—C1B—C11B—C12B	56.6 (3)
C10—C1—C11—C16	−56.7 (3)	C10B—C1B—C11B—C12B	−125.7 (3)
C2—C1—C11—C12	−54.3 (3)	C2B—C1B—C11B—C16B	−120.6 (3)
C10—C1—C11—C12	127.3 (3)	C10B—C1B—C11B—C16B	57.1 (3)
C16—C11—C12—C13	−0.1 (4)	C16B—C11B—C12B—C13B	1.1 (3)
C1—C11—C12—C13	176.1 (2)	C1B—C11B—C12B—C13B	−176.2 (2)
C11—C12—C13—C14	−0.8 (4)	C11B—C12B—C13B—C14B	0.2 (3)
C11—C12—C13—C17	178.6 (2)	C11B—C12B—C13B—C17B	−179.0 (2)
C12—C13—C14—C15	1.0 (4)	C12B—C13B—C14B—C15B	−1.1 (3)
C17—C13—C14—C15	−178.4 (2)	C17B—C13B—C14B—C15B	178.2 (2)
C13—C14—C15—C16	−0.3 (4)	C13B—C14B—C15B—C16B	0.5 (3)
C13—C14—C15—C18	−178.6 (2)	C13B—C14B—C15B—C18B	178.7 (2)
C14—C15—C16—C11	−0.7 (4)	C14B—C15B—C16B—C11B	0.8 (4)
C18—C15—C16—C11	177.6 (2)	C18B—C15B—C16B—C11B	−177.3 (2)
C12—C11—C16—C15	0.9 (4)	C12B—C11B—C16B—C15B	−1.6 (4)
C1—C11—C16—C15	−175.3 (2)	C1B—C11B—C16B—C15B	175.7 (2)
C14—C13—C17—O2	174.1 (2)	C14B—C13B—C17B—O2B	−174.2 (2)
C12—C13—C17—O2	−5.3 (4)	C12B—C13B—C17B—O2B	5.0 (3)
C14—C13—C17—O1	−5.9 (3)	C14B—C13B—C17B—O1B	5.9 (3)
C12—C13—C17—O1	174.6 (2)	C12B—C13B—C17B—O1B	−174.9 (2)
C16—C15—C18—O3	−173.5 (2)	C16B—C15B—C18B—O3B	173.8 (2)
C14—C15—C18—O3	4.8 (4)	C14B—C15B—C18B—O3B	−4.4 (4)
C16—C15—C18—O4	5.4 (3)	C16B—C15B—C18B—O4B	−5.9 (3)
C14—C15—C18—O4	−176.3 (2)	C14B—C15B—C18B—O4B	176.0 (2)
C10A—C1A—C2A—C3A	−2.7 (3)	C10C—C1C—C2C—C3C	1.5 (4)
C11A—C1A—C2A—C3A	174.1 (2)	C11C—C1C—C2C—C3C	−177.6 (2)
C1A—C2A—C3A—C4A	−0.3 (4)	C1C—C2C—C3C—C4C	1.2 (4)
C2A—C3A—C4A—C5A	2.8 (4)	C2C—C3C—C4C—C5C	−2.5 (4)
C3A—C4A—C5A—C6A	177.7 (2)	C3C—C4C—C5C—C6C	−177.8 (2)
C3A—C4A—C5A—C10A	−2.1 (3)	C3C—C4C—C5C—C10C	1.1 (4)
C4A—C5A—C6A—C7A	−178.3 (2)	C4C—C5C—C6C—C7C	177.9 (2)
C10A—C5A—C6A—C7A	1.5 (4)	C10C—C5C—C6C—C7C	−1.1 (3)
C5A—C6A—C7A—C8A	0.8 (4)	C5C—C6C—C7C—C8C	−1.1 (4)
C6A—C7A—C8A—C9A	−1.8 (4)	C6C—C7C—C8C—C9C	1.7 (4)
C7A—C8A—C9A—C10A	0.3 (4)	C7C—C8C—C9C—C10C	−0.1 (4)
C8A—C9A—C10A—C1A	179.8 (2)	C8C—C9C—C10C—C5C	−2.0 (3)
C8A—C9A—C10A—C5A	2.0 (3)	C8C—C9C—C10C—C1C	−179.9 (2)
C2A—C1A—C10A—C9A	−174.5 (2)	C6C—C5C—C10C—C9C	2.6 (3)
C11A—C1A—C10A—C9A	8.7 (3)	C4C—C5C—C10C—C9C	−176.4 (2)
C2A—C1A—C10A—C5A	3.3 (3)	C6C—C5C—C10C—C1C	−179.5 (2)
C11A—C1A—C10A—C5A	−173.5 (2)	C4C—C5C—C10C—C1C	1.5 (3)
C4A—C5A—C10A—C9A	176.9 (2)	C2C—C1C—C10C—C9C	175.0 (2)
C6A—C5A—C10A—C9A	−2.8 (3)	C11C—C1C—C10C—C9C	−5.9 (3)
C4A—C5A—C10A—C1A	−1.0 (3)	C2C—C1C—C10C—C5C	−2.8 (3)
C6A—C5A—C10A—C1A	179.3 (2)	C11C—C1C—C10C—C5C	176.3 (2)
C2A—C1A—C11A—C16A	56.6 (3)	C2C—C1C—C11C—C16C	−54.5 (3)
C10A—C1A—C11A—C16A	−126.5 (3)	C10C—C1C—C11C—C16C	126.4 (2)
C2A—C1A—C11A—C12A	−120.0 (3)	C2C—C1C—C11C—C12C	121.9 (3)
C10A—C1A—C11A—C12A	56.9 (3)	C10C—C1C—C11C—C12C	−57.2 (3)
C16A—C11A—C12A—C13A	−1.1 (4)	C16C—C11C—C12C—C13C	1.2 (3)
C1A—C11A—C12A—C13A	175.6 (2)	C1C—C11C—C12C—C13C	−175.3 (2)
C11A—C12A—C13A—C14A	1.2 (4)	C11C—C12C—C13C—C14C	−0.5 (3)
C11A—C12A—C13A—C17A	−177.8 (2)	C11C—C12C—C13C—C17C	177.6 (2)
C12A—C13A—C14A—C15A	0.0 (4)	C12C—C13C—C14C—C15C	−0.8 (3)
C17A—C13A—C14A—C15A	179.1 (2)	C17C—C13C—C14C—C15C	−179.0 (2)
C13A—C14A—C15A—C16A	−1.3 (4)	C13C—C14C—C15C—C16C	1.5 (3)
C13A—C14A—C15A—C18A	178.5 (2)	C13C—C14C—C15C—C18C	−178.6 (2)
C12A—C11A—C16A—C15A	−0.1 (4)	C12C—C11C—C16C—C15C	−0.5 (3)
C1A—C11A—C16A—C15A	−176.8 (2)	C1C—C11C—C16C—C15C	176.0 (2)
C14A—C15A—C16A—C11A	1.4 (4)	C14C—C15C—C16C—C11C	−0.9 (3)
C18A—C15A—C16A—C11A	−178.5 (2)	C18C—C15C—C16C—C11C	179.2 (2)
C12A—C13A—C17A—O1A	172.8 (2)	C12C—C13C—C17C—O1C	−174.8 (2)
C14A—C13A—C17A—O1A	−6.2 (4)	C14C—C13C—C17C—O1C	3.3 (4)
C12A—C13A—C17A—O2A	−6.2 (3)	C12C—C13C—C17C—O2C	4.4 (3)
C14A—C13A—C17A—O2A	174.8 (2)	C14C—C13C—C17C—O2C	−177.5 (2)
C16A—C15A—C18A—O4A	5.8 (4)	C14C—C15C—C18C—O4C	174.9 (2)
C14A—C15A—C18A—O4A	−174.0 (2)	C16C—C15C—C18C—O4C	−5.2 (3)
C16A—C15A—C18A—O3A	−174.2 (2)	C14C—C15C—C18C—O3C	−5.1 (3)
C14A—C15A—C18A—O3A	6.0 (3)	C16C—C15C—C18C—O3C	174.8 (2)

### Hydrogen-bond geometry (Å, º)

**Table:**

*D*—H···*A*	*D*—H	H···*A*	*D*···*A*	*D*—H···*A*
O1—H1···O1*W*^i^	0.84	1.72	2.559 (2)	175
O1*B*—H1*B*···O3*W*	0.84	1.74	2.573 (2)	168
O2*A*—H2*A*···O4*A*^ii^	0.84	1.82	2.583 (2)	151
O2*C*—H2*C*···O4*C*^ii^	0.84	1.81	2.584 (2)	152
O3*A*—H3*A*···O2*W*^iii^	0.84	1.73	2.565 (3)	174
O3*C*—H3*C*···O4*W*	0.84	1.72	2.561 (2)	176
O4—H4···O2^iii^	0.84	1.81	2.591 (2)	154
O4*B*—H4*B*···O2*B*^iii^	0.84	1.81	2.578 (2)	150
O1*W*—H1*W*1···O1*H*^iv^	0.85 (3)	1.92 (3)	2.716 (3)	155 (3)
O1*W*—H2*W*1···O3^v^	0.85 (3)	2.04 (3)	2.858 (2)	162 (3)
O2*W*—H1*W*2···O1*G*^vi^	0.85 (3)	1.91 (3)	2.742 (3)	168 (3)
O2*W*—H2*W*2···O1*A*	0.85 (2)	2.03 (2)	2.879 (2)	174 (3)
O3*W*—H1*W*3···O1*G*^vii^	0.85 (2)	1.85 (2)	2.680 (3)	165 (3)
O3*W*—H2*W*3···O3*B*^ii^	0.85 (2)	1.98 (2)	2.824 (2)	170 (3)
O4*W*—H1*W*4···O1*H*^vii^	0.85 (2)	1.84 (2)	2.656 (3)	162 (3)
O4*W*—H2*W*4···O1*C*^iii^	0.86 (3)	1.96 (3)	2.808 (2)	172 (3)

Symmetry codes: (i) *x*−1, *y*+1, *z*; (ii) *x*, *y*+1, *z*; (iii) *x*, *y*−1, *z*; (iv) *x*, *y*, *z*+1; (v) *x*+1, *y*, *z*; (vi) *x*−1, *y*, *z*+1; (vii) *x*−1, *y*, *z*.
